# Production of Gamma-Aminobutyric Acid from Lactic Acid Bacteria: A Systematic Review

**DOI:** 10.3390/ijms21030995

**Published:** 2020-02-03

**Authors:** Yanhua Cui, Kai Miao, Siripitakyotin Niyaphorn, Xiaojun Qu

**Affiliations:** 1 Department of Food Science and Engineering, School of Chemistry and Chemical Engineering, Harbin Institute of Technology, Harbin 150090, China; miaokai@163.com (K.M.);; 2 Institute of Microbiology, Heilongjiang Academy of Sciences, Harbin 150010, China; qvxiaojun@163.com

**Keywords:** gamma-aminobutyric acid, lactic acid bacteria, genetic engineering, physiology, co-culture engineering

## Abstract

Gamma-aminobutyric acid (GABA) is widely distributed in nature and considered a potent bioactive compound with numerous and important physiological functions, such as anti-hypertensive and antidepressant activities. There is an ever-growing demand for GABA production in recent years. Lactic acid bacteria (LAB) are one of the most important GABA producers because of their food-grade nature and potential of producing GABA-rich functional foods directly. In this paper, the GABA-producing LAB species, the biosynthesis pathway of GABA by LAB, and the research progress of glutamate decarboxylase (GAD), the key enzyme of GABA biosynthesis, were reviewed. Furthermore, GABA production enhancement strategies are reviewed, from optimization of culture conditions and genetic engineering to physiology-oriented engineering approaches and co-culture methods. The advances in both the molecular mechanisms of GABA biosynthesis and the technologies of synthetic biology and genetic engineering will promote GABA production of LAB to meet people’s demand for GABA. The aim of the review is to provide an insight of microbial engineering for improved production of GABA by LAB in the future.

## 1. Introduction

Gamma-aminobutyric acid (GABA) is a four-carbon free amino acid that is produced from l-glutamic acid by glutamate decarboxylase (GAD) and is widely present in microorganisms, plants, and animals [1]. It is well known that GABA is considered a bioactive component with multiple physiological functions [2]. GABA acts as a major inhibitory neurotransmitter that sends chemical messages in the mammalian central nervous system [3]. Moreover, GABA also plays an important role in behavior, cognition, and the body’s response to stress. GABA as a supplement is involved in improving sleeplessness and depression [4,5], enhancing immunity [5], relieving anxiety and menopausal syndrome [6], regulating blood pressure [7], fighting obesity [8], and improving visual cortical function [9].

At present, food containing GABA cannot meet people’s needs because of the low content of GABA. Microorganisms are an important source of GABA. Up to now, it has been confirmed that many types of microorganisms can synthesize GABA, including yeast, fungi, and bacteria [10,11] (Supplementary Table S1). Microorganisms grow faster than plants, do not need much space for cultivation, and are eco-friendly for consumers. Furthermore, it is easy to control production of microorganisms.

Lactic acid bacteria (LAB) are widely used in the foods industry, particularly in manufacturing fermented foods for many centuries [12]. Because of the generally recognized as safe (GRAS) status of LAB and their high application potential in the fermentation industry, GABA-producing LAB have received extensive attention in recent years. A large number of GABA-producing LAB were isolated from fermented food and used in the manufacturing of naturally health-oriented foods enriched with GABA. In view of the food-grade nature of LAB and their potential as a functional food, this paper will focus on the GABA-producing LAB.

In the present review, we will describe GABA-producing LAB species and the biosynthetic pathway of GABA in LAB, with particular emphasis on the diversity of the GAD system of LAB. Furthermore, GABA production enhancement strategies are discussed, from optimization of culture conditions and genetic engineering to physiology-oriented engineering approaches and co-culture methods.

## 2. GABA-Producing LAB Species

LAB are important GABA-producing microorganisms because of their various production characteristics and probiotic effects. A number of LAB strains with GABA-producing capability have been isolated from traditional fermented foods such as cheese, kimchi, paocai, yoghurt, and fermented soy beans and so on (Supplementary Table S2).

The genus Lactobacillus has abundant GABA-producing species, including Lactobacillus brevis [13,14,15,16,17,18,19], Lactobacillus buchneri [20,21], Lactobacillus delbrueckii subsp. bulgaricus [17,22], Lactobacillus fermentum [23,24], Lactobacillus helveticus [25], Lactobacillus paracasei [17,26], Lactobacillus plantarum [17,26,27], and so on. Furthermore, some Streptococcus thermophilus and Lactococcus lactis strains display GABA production abilities, which are the best candidates for production of GABA-rich milk products [17,19,26,28]. In recent years, it has been found that some species from the genera Enterococcus, Leuconostoc, Pediococcus, Propionibacterium, and Weissella are capable of producing GABA (Supplementary Table S2).

l-glutamate-rich fermented foods are important isolation sources of GABA-producing LAB. Cheese contains a large number of caseins that could produce plenty of l-glutamate. *Lb. buchneri*, *Lb. brevis*, *Lb. paracasei*, *Lb. plantarum*, *Lb. delbrueckii* subsp. *bulgaricus*, and *Lc. lactis* strains isolated from traditional or commercial cheeses have been shown to produce GABA [17,19,20,26]. Generally, an acid environment is beneficial for the growth of GABA-producing LAB, such as in Korean kimchi and Chinese paocai.

The GABA production capacity of different species is greatly varied. A large number of studies have shown that *Lb. brevis* can produce a high yield of GABA compared with other LAB species (Supplementary Table S2). Up to now, the highest yield of GABA by *Lb. brevis* is 205 g/L [18]. At the same time, various strains of one species have obvious differences in GABA productivity (Supplementary Table S2). Different strains of *Lb. brevis* produce GABA in the range from 15.0 mg/L to 205 g/L [17,18]. Similarly, different *Lb. plantarum* strains isolated from various cheeses have diverse GABA productivity [17,26,27].

## 3. Biosynthesis of GABA in LAB

### 3.1. Biosynthetic Pathway of GABA in LAB

**Figure 1:**
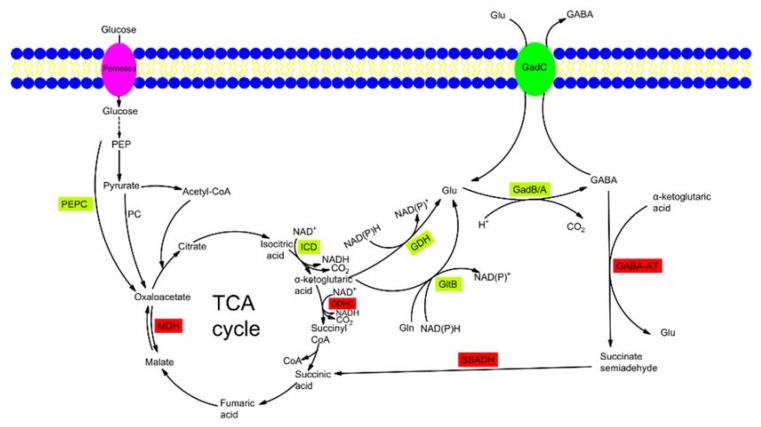
The biosynthetic pathway of GABA by microbes. GABA-AT, GABA aminotransferase; GadB/GadA, glutamate decarboxylase; GadC, glutamate: γ-aminobutyrate antiporter; GDH, l-glutamate dehydrogenase; GltB, glutamate synthase; Icd, isocitrate dehydrogenase; MDH, malate dehydrogenase; ODHC, 2-oxoglutarate dehydrogenase complex; PC, pyruvate carboxylase; PEP, phosphoenolpyruvate; PEPC, phosphoenolpyruvate carboxylase; SSADH, succinate semialdehyde dehydrogenase; TCA cycle, tricarboxylic acid cycle. The expression of enzymes in green font will increase GABA production, and the expression of enzymes in red font will decrease GABA production.

The biosynthesis of GABA by microorganisms is performed by the glutamic acid decarboxylase (GAD, EC 4.1.1.15) system, which is composed of the GAD enzyme (encoded by *gadA* or *gadB*) and glutamate/GABA antiporter GadC [29,30,31,32,33]. The biosynthetic pathway of GABA by microbes is shown in Figure 1.

l-glutamate is transported into a cell through GadC. The decarboxylation of l-glutamate is catalyzed by GAD with cofactor pyridoxal-5′-phosphate (PLP), and leads to the formation of GABA and release of CO_2_ as byproduct (Figure 1). Finally, the decarboxylated product GABA is exported to the extracellular matrix by GadC [29]. As the precursor of l-glutamate, a-ketoglutarate is synthesized from glucose via the glycolysis pathway and part of the tricarboxylic acid (TCA) cycle, and then it is converted into l-glutamate by l-glutamate dehydrogenase (GDH, EC 1.4.1.4). The 2-oxoglutarate dehydrogenase complex (ODHC) is the key enzyme of the TCA cycle and acts at the branching point of metabolic flux between l-glutamate synthesis and energy supply. Moreover, it competes with GDH for the substrate a-ketoglutarate.

In some bacteria, such as *Escherichia coli* and *Listeria monocytogenes*, the molecular mechanisms of GABA degradation have been clarified [34,35]. GABA is degraded to succinic semialdehyde (SSA) by the major GABA-degradative enzyme GABA aminotransferase (GABA-AT, EC 2.6.1.19), and then it later converts to succinic acid at catalysis of succinate semialdehyde dehydrogenase (SSADH, EC 1.2.1.16) for entry into the TCA cycle.

The GABA-AT encoding gene *gadT* has been identified in the some species of LAB, such as *Lb. fermentum*, *Lactobacillus frumenti*, *Lactobacillus gastricus*, *Lactobacillus gorilla*, *Lactobacillus mucosae*, *Lactobacillus oris*, *Lb. plantarum* [36], *Lactobacillus pontis*, *Lactobacillus reuteri* [37], *Lactobacillus similis*, *Lactobacillus vaginalis*, *Leuconostoc citreum* [38], I subsp. *gasicomitatum* [39], *Leuconostoc kimchii* [40], *Leuconostoc mesenteroides* subsp. *mesenteroides* [41], *Oenococcus oeni* [41], and so on. Recently, it has been found that GABA production of *Lb. plantarum* is obviously increased in the fermentation with MSG [27]. At the same time, the expression of gene *gadT* was down-regulated by a factor of 0.26 times, indicating that the activity of GABA-AT was inhibited. The results show that GABA-AT activity has a direct relationship with the GABA production.

### 3.2. GAD System of LAB

**Figure 2:**
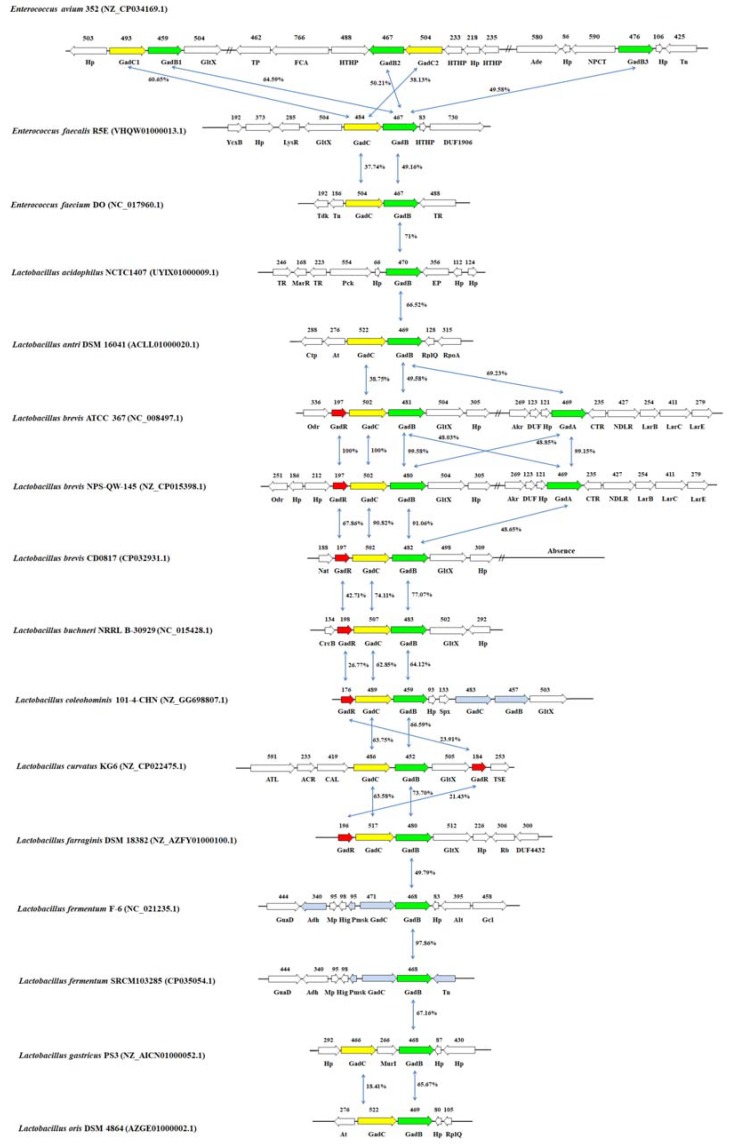

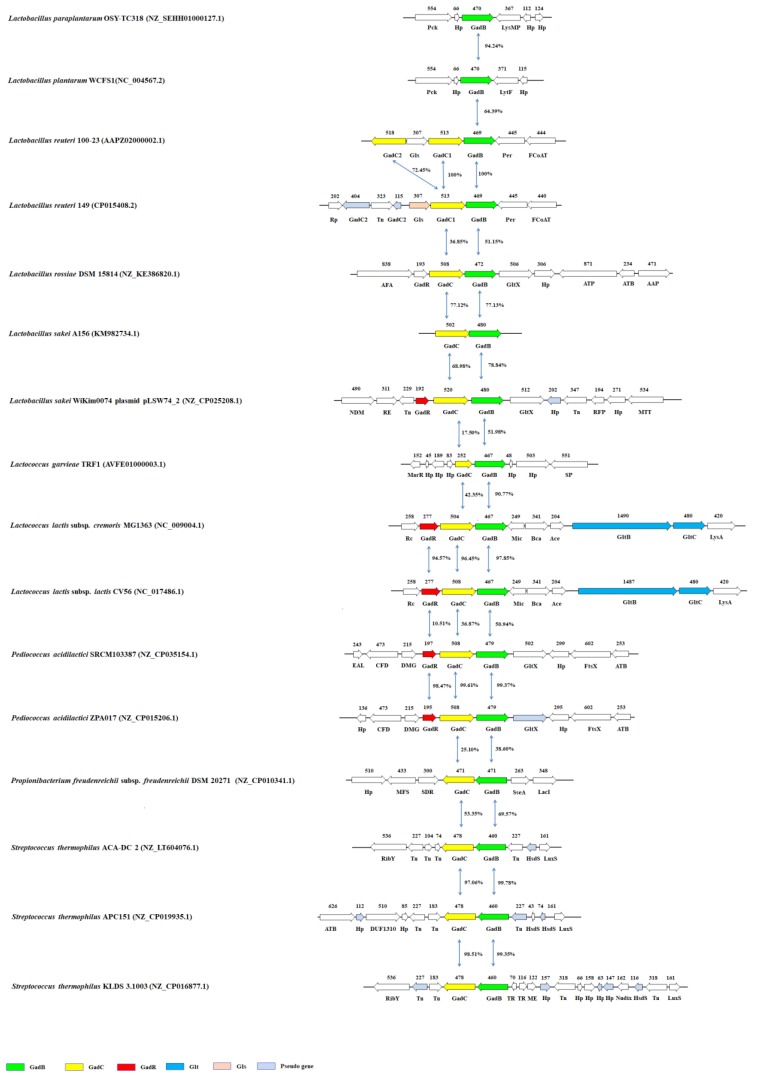
Genetic organization of the glutamate decarboxylase gene clusters among some lactic acid bacteria (LAB) strains. The number of amino acids within each encoded protein is shown above the corresponding protein. AAP, amino acid permease; Ace, sugar O-acetyltransferase; ACR, amino acid racemase; Ade, adenine deaminase; Adh, zinc-dependent alcohol dehydrogenase family protein; AFA, AAA family ATPase; Akr, aldo/keto reductase; Alt, PLP-dependent aminotransferase family protein; At, metN2, ABC transporter, ATP-binding protein; ATB, ABC transporter ATP-binding protein; ATL, aspartate—tRNA ligase; ATP, ABC transporter permease; Bca, branched-chain amino acid aminotransferase; CAL, carboxylate—amine ligase; CFD, C69 family dipeptidase; CrcB, fluoride efflux transporter; Ctp, cobalt ABC transporter, ATP-binding protein; CTR, Crp/Fnr family transcriptional regulator; DMG, DNA-3-methyladenine glycosylase; DUF, DUF1722 domain-containing protein; DUF1310, DUF1310 family protein; DUF1906, DUF1906 domain-containing protein; DUF4432, DUF4432 family protein; EAL, EAL domain-containing protein; EP, extracellular protein; FCA, formate C-acetyltransferase; FCoAT, formyl-CoA transferase; FtsX, FtsX-like permease family protein; GadB/GadA, glutamate decarboxylase; GadC/GadC1/GadC2, glutamate:gamma-aminobutyrate antiporter; GadR, transcriptional regulator; Gcl, glutamate-cysteine ligase; Gls, glutaminase; GltB, glutamate synthase large subunit; GltC, glutamate synthase subunit beta; GltX, glutamate-tRNA ligase; GuaD, guanine deaminase; Hig, HigA family addiction module antidote protein; Hp, hypothetical protein; HsdS, restriction endonuclease subunit S; HTHP, helix-turn-helix domain-containing protein; LacI, LacI family transcriptional regulator; LarB, nickel pincer cofactor biosynthesis protein; LarC, nickel pincer cofactor biosynthesis protein; LarE, ATP-dependent sacrificial sulfur transferase; LuxS, S-ribosylhomocysteine lyase; LysA, diaminopimelate decarboxylase; LysMP, LysM peptidoglycan-binding domain-containing protein; LysR, LysR family transcriptional regulator; LytF, gamma-d-glutamate-meso-diaminopimelate muropeptidase; MarR, MarR family transcriptional regulator; ME, ImmA/IrrE family metallo-endopeptidase; MFS, MFS transporter; Mic, mechanosensitive ion channel; Mp, membrane protein; MTT, class I SAM-dependent methyltransferase; MurI, glutamate racemase; Nat, *N*-acetyltransferase; NDLR, nickel-dependent lactate racemase; NDM, N-6 DNA methylase; NPCT, Na/Pi cotransporter family protein; Nudix, NUDIX domain-containing protein; Odr, oxidoreductase; Pck, phosphoenolpyruvate carboxykinase; Per, NCS2 family permease; Pmsk, plasmid maintenance system killer; Rb, ribokinase; Rc, ribonuclease HII; RE, restriction endonuclease; RFP, recombinase family protein; RibY, ribonuclease Y; Rp, 30S ribosomal protein S4; RplQ, ribosomal protein L17; RpoA, DNA-directed RNA polymerase, alpha subunit; SDR, SDR family oxidoreductase; SP, sulfate permease; Spx, Spx/MgsR family RNA polymerase-binding regulatory protein; SseA, sulfurtransferase; Tdk, thymidine kinase; Tn, transposase; TP, l,d-transpeptidase family protein; TR, transcriptional regulator; TSE, threonine/serine exporter family protein; YcxB, YcxB family protein.

The GAD system is present in various LAB species, including genera *Enterococcus*, *Lactobacillus*, *Lactococcus*, *Pediococcus*, *Propionibacterium*, and *Streptococcus* (Figure 2). Its genetic organization shows a high variability. Although some LAB species have GABA-producing ability, such as *Lactobacillus casei*, *Lb. paracasei*, *Leuconostoc mesenteroides*, and *Weissella hellenica*, there is little information about the related genes of GABA production in the genomes of these species [17,42,43,44].

Most GAD systems of LAB species locate in the chromosome; however, that of *Lactobacillus sakei* WiKim0074 is from its plasmid. A glutamate-tRNA ligase gene *gltX* is found upstream of the *gadB-gadC* operon of some species. Furthermore, glutamate synthases encoding genes *gltB* and *gltC* are located near the *gadB-gadC* gene in *Lc. lactis* [45,46]. It was speculated that these glutamate metabolism related genes could facilitate GABA production.

For different strains of the same species, genetic organization of the GAD system also presents variability. For example, the *gadB-gadC* genes are flanked by transposases elements in *S. thermophilus*. In *S. thermophilus* strains ACA-DC 2, B59671, and TH1435, S-ribosylhomocysteine lyase and ribonuclease Y locate, respectively, upstream and downstream of the *gadB-gadC* operon [47,48,49]. However, for strains ND03 and APC151, DUF1310 family protein and ABC transporter ATP-binding protein locate downstream of *gadB-gadC* [50,51]. Furthermore, the genetic organization of *gadB-gadC* in strain KLDS 3.1003 is very special, with an additional ca. 7 kb polynucleotide stretch including transposase, NUDIX domain-containing protein, and XRE family transcriptional regulator elements between S-ribosylhomocysteine lyase and GadB [52].

**Figure 3:**
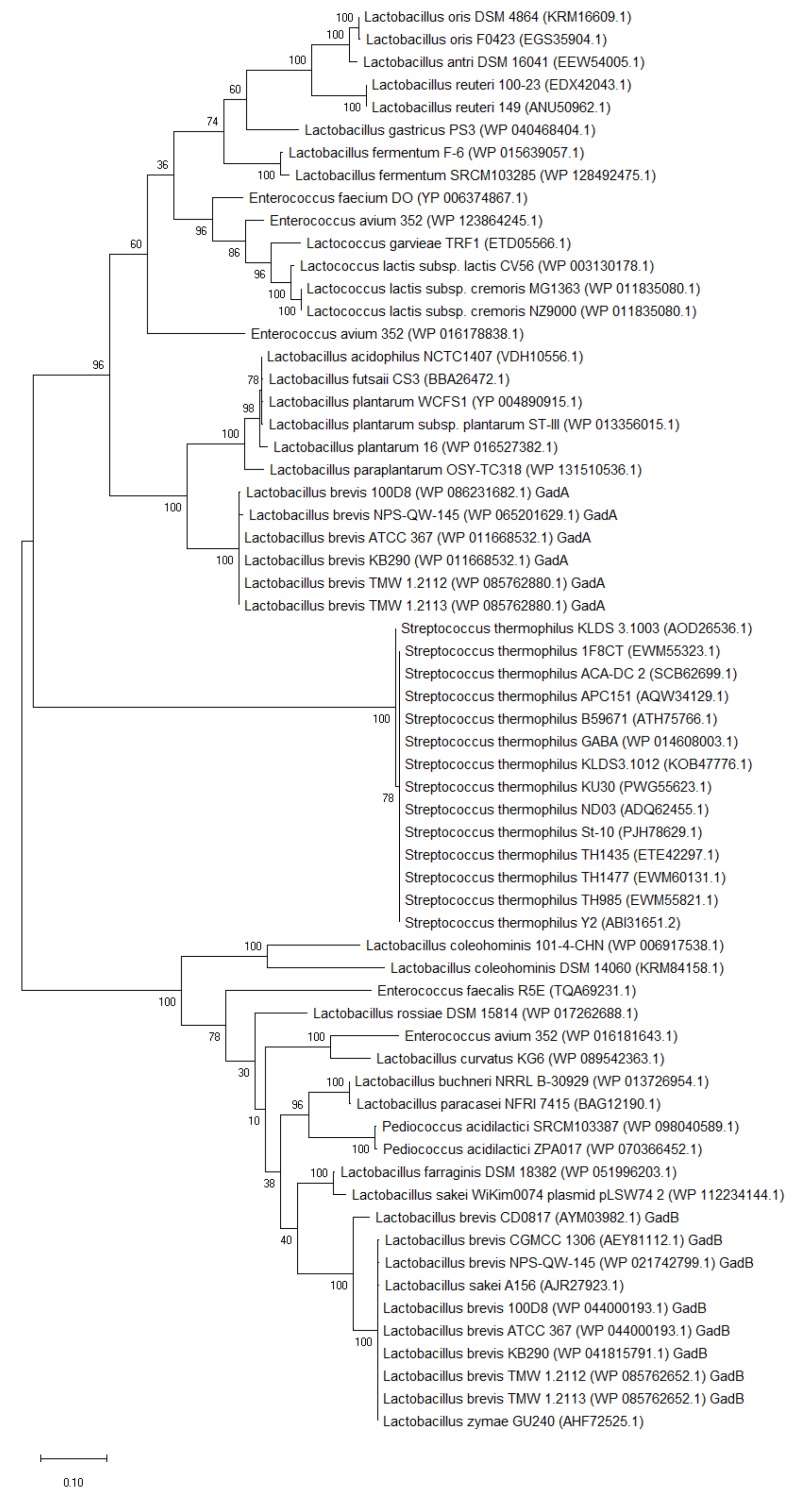
Phylogenetic tree (maximum-likelihood method) based on amino acid sequences of GAD from LAB. Figure was generated from MEGA (version X 10.1) after ClustalW alignment of GADs. The GenBank accession number of GAD is indicated in the braces.

GAD is the key enzyme for the bioconversion of GABA and localizes in the cytoplasm. The strains with the GAD gene are able to synthesize GABA. Generally, most GABA-producing LAB species have a GAD-encoding gene (Figure 2 and Figure 3). Interestingly, *Enterococcus avium* 352 has three GADs, and most *Lb. brevis* strains contain two distinct GAD encoding genes [1,30,53,54].

The phylogenetic tree analysis shows that the amino acid sequences of GADs from different LAB species are highly conserved in the same species (Figure 3). These GADs belong to the pyridoxal-5′-phosphate (PLP)-dependent decarboxylase super-family, and they possess a highly conserved lysine residue (Lys_278_ or Lys_279_), which is essential for the binding of the PLP, as well as the active site residues (Thr_214_ and Asp_245_; or Thr_215_ and Asp_246_) that promote decarboxylation.

It is worth noting that the names of two GAD-encoding genes in *Lb. brevis* are different in different studies. In this review, the classification of GADs from *Lb. brevis* was based on previous studies [30]. One *gad* gene (*gadB*) is located in an operon with *gadC*. However, the other *gad* gene (*gadA*) has a far genetic distance with those from other LAB and is located separately from the other *gad* genes [1,16,30,54]. GadA and GadB have only about 48% identity in amino acid sequences; however, the PLP-binding domain where the active site residues of both GADs are highly conserved. GAD activity was mainly contributed by GadB in *Lb. brevis* in the previous study [30].

Recently, it was found that a high GABA-producing *Lb. brevis* CD0817 contained only a *gadB-gadC* operon and was absent of *gadA* gene [55]. What is more, there are obvious differences between GadB of CD0817 and those in the other GABA-producing *Lb. brevis* strains, with only 91% of amino acid sequence identity. The results further demonstrated that GadB contributed to the main GAD activity [55]. *gadA* does not have a close relationship with the accumulation of GABA. It was speculated that this distinctive GAD system contributed to the high GABA productivity of *Lb. brevis* CD0817.

GADs have been isolated from several LAB species, and their biochemical properties have been characterized (Table 1). Although the characteristics of GADs from different LAB species and strains are very different, most LAB GADs exhibit optimal activity at pH 4.0–5.0 (Table 1). Moreover, GADs significantly lose activity at near-neutral pH (pH 7.0).

Some studies suggested that the C-terminal region of GAD is involved in the pH dependence of catalysis [66,71]. The C-terminally truncated mutant of *Lb. plantarum* GAD showed a pronounced catalytic activity in a broad pH range of 4.0–8.0 [66]. Compared with the wild type, a GAD mutant with a 14C-terminal residue truncation from *Lb. brevis* CGMCC 1306 exhibited an extended enzymatic activity toward near-neutral pH [71]. Therefore, the C-terminal GAD could as an effective target region for improving the pH dependence of GAD activity. The optimal temperatures of GADs are varied in different species of LAB, ranging from 30 to 60 °C (Table 1). *S. thermophillus* Y2 presented an increased GAD activity at 34–37 °C, but the GAD activity decreased when temperature continued to increase from 37 to 46 °C [28].

The crystal structure of GAD from *Lb. brevis* CGMCC 1306 has been successfully solved [72]. It is different from the GAD of *E. coli*, which contains six repetitive subunits. It consists of two repetitive subunits composed of a conserved lysine residue, Lys_279_, that binds to coenzyme PLP. Furthermore, some key residues of GAD (Ser_126_, Ser_127_, Cys_168_, Ile_211_, Ser_276_, His_278_, and Ser_321_) have been determined, which play essential roles in anchoring the PLP cofactor inside the active site and supporting GAD catalytic reactivity. The flexible loop (Tyr_308_-Glu_312_), which is positioned near the substrate-binding site, is involved in the catalytic reaction, and the conserved residue Tyr_308_ plays a critical role in decarboxylation of l-Glu.

GAD structural information will help us to understand its catalytic mechanism and the structure-function relationship. Moreover, it offers a new direction for improving GAD activity and stability by means of rational or semirational methods. Compared with the wild-type GAD of *Lb. brevis* CGMCC 1306, the mutant T215A, which the mutagenesis site is around the putative substrate pocket, showed a 1.6-fold improvement in catalytic efficiency [72].

The *gadC* gene has been identified to encode a GABA antiporter, which is involved in the GABA export and glutamate import [73]. Generally, LAB species contain a GABA antiporter, but *Lb. fermentum* possesses a GAD that is not accompanied by a GABA antiporter; *Lb. reuteri* has two GABA antiporters (Figure 2).

The GadR is a positive transcriptional regulator that can activate the *gadB* gene and control the GABA conversion in *Lc. lactis* and *Lb. brevis* [74,75]. In *Lc. lactis*, GadR expression is chloride and glutamate independent [74]. The study showed that GadR has a close relationship with the GABA production, and hyper expression of GadR could increase the GABA productivity [75]. At the same time, GadR could improve the acid resistance of the strain [75]. Some potential transcriptional regulators of GABA production were found in several LAB species such as *Lb. buchneri*, *Lactobacillus coleohominis*, *Lactobacillus curvatus*, *Lactobacillus farraginis*, *Lb. sakei*, *Lactococcus lactis* subsp. *cremoris*, *Lactococcus lactis* subsp. *lactis*, and *Pediococcus acidilactici* (Figure 2). Generally, *gadR* locates immediately upstream of the operon *gadB-gadC*. It is different from the high identities of GadB of different LAB species; these GadRs present extremely low identities in amino acids sequences (Figure 2).

In a word, the GAD system of LAB has strain specificity. A great number of researchers indicated that strains with identical GAD systems showed different GABA productivities. Therefore, GABA production is a complicated process.

## 4. Improvement of the Production of GABA of LAB

A number of LAB species have shown strain-specific capability in the synthesis of GABA. A number of studies have demonstrated that culture conditions play an important role in GABA production. Therefore, developing higher-yield strains (e.g., by strain selection and breeding, mutagenesis, or genetic manipulation) and optimizing fermentation conditions are two approaches for improving GABA yield. At the same time, fermented food with GABA-producing LAB is a good choice because GABA extraction is eliminated.

### 4.1. Optimization of Culture Conditions

The GABA production capacity of strains is significantly affected by culture conditions. A large number of studies have been performed to improve GABA yield via optimizing fermentation conditions, such as optimizing the initial pH value of culture medium, fermentation temperature, fermentation time, l-glutamic acid concentration, PLP, media additives, carbon source, nitrogen source, and so on [14,76,77,78].

The pH value is a key factor for GABA biosynthesis by LAB; it not only influences the growth of bacteria, but also affects the GAD activity [15,21,28,79,80]. Some studies have shown that the initial pH of fermentation medium affected GABA synthesis [15,79]. *Lb. brevis* NCL912 could produce the maximum GABA level at initial pH 5.0 [15]. Compared with initial pH 4.0 and pH 6.0, GABA production of *Lb. paracasei* NFRI 7415 was significantly enhanced, reaching 210 mM at initial pH 5.0 [80]. The optimal initial pH is also 5.0 for GABA synthesis of *Lb. buchneri* [21]. However, *Lb. brevis* GABA100 achieved the highest GABA yield at initial pH 3.5 during the fermentation of black raspberry juice [79]. Therefore, the optimal conditions of fermenting microorganisms vary according to the different properties of GADs, with optimal pH ranging from pH 3.5–5.0.

Maintaining low pH (about 5) is necessary for effective GABA production [28,80]. During the fermentation of LAB, the pH value of the culture continuously decreases. GABA production of *S. thermophilus* Y2 was significantly increased by means of adjusting the pH of culture medium to pH 4.5 every 12 h by adding NaOH or HCl after free incubation for 24 h [28]. The results have demonstrated that keeping an optimal pH during GABA production is an effective way to enhance GABA yield.

Culture temperature is an important factor for GABA production and growth of bacteria. The fermentation of *Lb. brevis* strain GABA 100 at 30 °C showed higher production of GABA in black raspberry juice than those at 25 and 37 °C [79]. The optimal temperature is 37 °C for GABA production of *Lb. brevis* CRL 1942 [77].

GAD activity is the key factor to determine the GABA yield of a strain. It is not only affected by pH and temperature but also by l-glutamic acid and PLP. As the substrate of GAD, l-glutamic acid is an indispensable compound in the medium for the synthesis of GABA by LAB, as LAB cannot synthesize enough l-glutamic acid for GABA production. Generally, monosodium glutamate (MSG) is used in GABA production because it can produce l-glutamic acid by hydrolysis.

Increasing MSG is aimed to stimulate the production of GABA by GAD via the GABA shunt way. At the same time, some researchers have demonstrated that excessive MSG could inhibit cell growth and decrease GABA production [27,28,77]. The optimal concentrations of MSG are different for various microorganisms in GABA production. The GABA production of *S. thermophillus* Y2 has no obvious change at the range from 10 to 20 g/L MSG [28]. The optimal MSG content is 270 mM for GABA production of *Lb. brevis* CRL 1942 [77]. MSG significantly induced GABA production in *Lb. plantarum* CGMCC 1.2437^T^ during fermentation [27]. At conditions containing 100 mM L-MSG, the GABA yield was 721.35 mM, which was 7.7 times more than that without supplement MSG. Transcriptomic analysis revealed that MSG increased the expression of key enzymes of carbohydrate metabolism, fatty acid synthesis, and amino acid metabolism [27].

PLP can increase GAD activity by acting as a cofactor for the enzyme GAD. The effect of PLP varies with the time of the addition of PLP. It was found that PLP could greatly promote GABA production of *Lb. paracasei* at 10 or 100 μM concentrations in the initial culture medium [80]. Yang et al. (2008) investigated the effect of PLP on GABA production. A 0.02 mM PLP sample was added in medium at different culture times respectively [28]. The addition of PLP could enhance GABA synthesis. Especially, when PLP was added at 48 h of fermentation, the GABA yield was much higher than that of PLP addition at 0 and 24 h of fermentation. It was speculated that PLP could have easily been influenced by some metabolites and lost its function as coenzyme of GAD during the fermentation. Therefore, it may be more efficient to enhance GABA yield by the addition of PLP at 48 h.

### 4.2. Genetic Engineering

Genetic engineering is an important strategy to improve GABA bioconversion and enhance the yield of GABA through the directed modulation of metabolic pathways. The direct approach is to overexpress the key enzyme GAD. Additional GAD-encoding genes were heterologously or homologously overexpressed in model strains *E. coli*, *Corynebacterium glutamicum*, and LAB such as *Lb. sakei*, *Lb. plantarum*, *Bifidobacterium longum*, and so on [58,60,81,82,83,84]. The efficiency of GABA producers was obviously improved. A recombinant *C. glutamicum* was constructed by the co-expression of two GAD genes, *gadB1* and *gadB2*, from *Lb. brevis* Lb85 [85]. Compared with the *gadB1* or *gadB2* single-expressing strains, GABA production by the co-expressing strain increased more than two-fold, and up to 18.66 ± 2.11 g/L after 84 h of fermentation.

In addition to overexpression of the GAD gene, the other genes in the pathway of GABA synthesis are overproduced in order to increase GABA productivity of strain, such as the glutamate: GABA antiporter gene *gadC* and the regulator gene *gadR.* The GABA yield of the engineered *E. coli* increased 2.5-fold in the early culture period via the co-overexpression of *gad* and *gadC* [82]. *C. glutamicum* strain ATCC 13032 was genetically engineered to synthesize GABA by the introduction of the *gadB2*, *gadC*, and *gadR* complex from *Lb. brevis* Lb85, and the GABA yield was up to 2.15 g/L with 72 h [86].

Furthermore, enhanced GABA biosynthesis efficiency and GABA productivity have also been achieved by inactivation of competing pathways of the GABA production. GABA aminotransferase gene *gabT* redirects GABA into the TCA cycle and results in GABA degradation. When the genes *gadB* and *gadC* were co-overexpressed in the *gabT* mutant strain, a final GABA concentration increased from 5.09 to 5.46 g/L [87].

GABA production needs sufficient precursor (l-glutamate), but GABA-producing LAB cannot naturally synthesize high concentrations of this compound; therefore, the fermentation process for GABA production by LAB must supply exogenous l-glutamate. Some overproducing l-glutamate recombinant strains have been developed to provide l-glutamate [88,89]. The recombinant *E. coli* producing 0.92 g/L of GABA directly from 10 g/L of glucose was constructed by the introduction of a synthetic protein scaffold consisting of three enzymes (isocitrate dehydrogenase, glutamate synthase, and GAD) of the GABA pathway [88]. Moreover, the novel GABA production system has been engineered for GABA production based on glucose medium by the co-localization of GABA transporter GadC and GABA shunt enzymes (i.e., succinate semialdehyde dehydrogenase and GABA-AT) [89].

Additionally, *C. glutamicum* is used to produce GABA using endogenous l-glutamate because of its strong amino acid producing ability [90]. A recombinant *C. glutamicum* harboring *E. coli*-derived GAD GadB yielded 8 g/L of GABA without the addition of l-glutamate after 96 h at 30 °C [91]. The serine/threonine protein kinase G gene *pknG* controls the activity of 2-oxoglutarate dehydrogenase (ODH) in the TCA branch point leading to glutamate synthesis [92]. GadB was expressed in the *C. glutamicum* strain GAD *∆pknG* containing a deletion of the *pknG* gene in order to enhance GABA production by increasing the intracellular concentration of its precursor l-glutamate. The *C. glutamicum* strain GAD *∆pknG* is an efficient strain in the de novo biosynthesis of GABA using its own accumulated l-glutamate, its GABA yield is 2.29-fold higher than that of strain GAD [92].

It was also found that GABA production was significantly increased by improvement of l-glutamate supplement via deletion of the 2-oxoglutarate decarboxylase subunit gene *odhA* or pyruvate carboxylase gene *pyc* [93]. *C. glutamicum* recombinants of overexpression of the phosphoenolpyruvate carboxylase gene *ppc* and/or deletion of the malate dehydrogenase gene *mdh* were also constructed to increase the l-glutamate supplement by efficient oxaloacetate supply [94].

Generally, the optimal pH of GAD activity is about 4.0–5.0, which is not good for the growth of GABA-producing strains. To expand the active pH range of the GAD GadB1 from *Lb. brevis* Lb85, three excellent mutants—GadB1^E312S^, GadB1^T17I/D294G/Q346H^, and GadB1^T17I/D294G/E312S/Q346H^—were obtained by directed evolution and site-specific mutagenesis. The GAD of these mutants showed higher activity and catalytic efficiency at a near-neutral pH (6.0) [95].

In order to resolve the discrepancy of optimal pH between GAD activity and cell growth, recombinant *C. glutamicum* strains were constructed by expressing *E. coli* GAD mutants with an expanded pH range activity; it provided a balanced condition for cell growth and GABA production. The recombinant strain could efficiently synthesize GABA in a culture pH around 5–7, and it produced 5.89 ± 0.35 g/L GABA in GP_1_ medium at pH 7.0 under the P_H36_ promoter, which was 17-fold higher than that of *C. glutamicum* expressing wild-type *E. coli* GAD in the same conditions [96].

In a word, genetic engineering is a useful strategy to improve GAD activity.

### 4.3. Physiology-Oriented Engineering Strategy

LAB are usually faced with various environmental stresses during the fermentation process and industry application, including acid, cold, heat, drying, oxidative stress, and so forth [97,98]. In response to these challenges, LAB strains need not only excellent metabolic capabilities but also strong physiological robustness and environmental fitness [99,100,101]. Therefore, physiology-oriented engineering has become an important way to increase work efficiency of industrially useful strains by improving their physiological performances [101].

The decreasing pH of the environment is a key type of stress for cell growth during GABA production. The acid-resistance system of LAB includes a proton pump, neutralization processes, DNA and protein damage repair, and so forth [97]. As the common acid resistance pathway, F_o_F_1_-ATPase regulates the cytoplasmic pH by means of expelling intracellular protons and generating the proton motive force [102]. GABA biosynthesis is archived through the decarboxylation of glutamate in the cytoplasm, of which this process needs to consume intracellular protons. Therefore, GAD system can maintain the intracellular pH value of bacteria and plays an important role in the acid resistance of cell [103]. In order to shift the influx of protons toward the GAD system and increase GABA production, a F_o_F_1_-ATPase-deficient and GAD-overexpressed *Lb. brevis* strain NRA6 was constructed [101]. Compared with those of the wild-type strain, the GABA conversion rate and yield of NRA6 obviously increased and was up to 98.42% and 43.65 g/L [101].

During the fermentation process, LAB will produce H_2_O_2_, which has potentially toxic effects for cells and inhibits cell growth [98]. In order to improve the antioxidative property of strains, a heme-dependent catalase (CAT) encoding gene *katE* of *Lb. brevis* CGMCC1306 was overexpressed. The recombinant strain *Lb. brevis* CAT showed a marked increased survival (823-fold) in an oxidizing environment [104]. Furthermore, the GABA production ability of the engineered *Lb. brevis* CAT was obviously enhanced and reached 66.4 g/L.

The results offer references to enhance the GABA production level by means of improving the technological properties of strains with physiology-oriented engineering strategies.

### 4.4. The Co-Culture Engineering

At present, the use of more than one microorganism in the fermentation industry is popular because some substances produced by co-culture strains could improve each other’s growth [105,106]. For this reason, co-fermentation with different strains is a very important and promising way for the high productivity of GABA. Some reports have described the production of GABA by co-fermentation.

As common co-culturing strains, *S. thermophilus* IFO13957 and *Lb. delbrueckii* subsp. *bulgaricus* IAM1120 were co-fermented and significantly raised GABA levels in the medium up to 15 mM, while when two strains were cultured separately, *S. thermophilus* IFO13957 and *Lb. bulgaricus* IAM1120 produced GABA around 0.25 and 0.15 mM, respectively [107].

A large number of *Lb. brevis* strains with high GABA yields were identified. Generally, *Lb. brevis* is isolated from fermented vegetables and do not have proteolytic activity because of the absence of extracellular proteinase-encoding genes. In order to resolve the limited application of *Lb. brevis* in fermented milk, a co-culture approach was adopted [108,109]. Conventional dairy starters were used in the co-fermentation because of their high proteolytic activity. They can break down milk protein into peptides and offer sufficient nutrition for *Lb. brevis.* A GABA-producing *Lb. brevis* 877G strain was used to ferment milk with the protease producer *Lb. sakei* 795, resulting in a significant increase in the GABA production (22.51 mM) [108].

One study demonstrated the dairy starter *S. thermophilus* YI-B1 could promote the viability and GABA biosynthesis of *Lb. brevis* NPS-QW-145 in milk [109]. The maximum concentration of GABA was 314 mg/kg containing 2 g/L of MSG supplement. During the fermentation, *S. thermophilus* provided sufficient amino acids or peptides and released formic acid, folic acid, and fatty acids, which could increase the growth of the *Lactobacillus* strains. However, *Lb. bulgaricus* had no obvious effect on the GABA production of *Lb. brevis* in milk [109]. It was speculated that *Lb. bulgaricus* and *Lb. brevis* belonged to the same *Lactobacillus* genus and were competitive. It was noted that not all tested strains of *S. thermophilus* could improve the GABA yield of *Lb. brevis* 145. This result indicated that the promoting of effect depends on the strain specificity.

*Lb. brevis* GABA 100 isolated from Korean kimchi displayed high GABA-producing ability [79]. A co-cultivation of *Lb. brevis* GABA 100 and *Bifidobacterium bifidum* BGN4 increased the GABA production in the fermentation of *Gastrodia elata* [110]. It was suggested the enhancement of GABA was achieved by the combination, further decreased the culture pH, and provided a suitable pH environment compared with the single culture of *Lb. brevis* GABA 100.

A combination of *Le. mesenteroides* SM and *Lb. plantarum* K154 was used for the production of a water dropwort (*Oenanthe javanica* DC)-based functional fermented food rich in GABA [111]. *Le. mesenteroides* is a dominant bacterium at the start of kimchi fermentation, is capable of producing biopolymers and CO_2_, and provides a thick broth and an enhanced acidic growth environment for the GABA-producing strain *Lb. plantarum* K154.

TRSF flour (turmeric and roasted soybean flour) was fermented by the two microorganism strains *Bacillus subtilis* HA and *Lb. plantarum* K154. It was found that, in the final stage of fermentation, GABA accumulation from co-fermentation was higher, up to 1.78% more than that of the single-fermented system [112]. *B. subtilis* HA and *Lb. plantarum* EJ2014 were used for the two-step fermentation of *Cucurbita moschata*, which is a pumpkin species rich in fiber, minerals, and carotenoids [113]. *B. subtilis* HA starter was inoculated in pumpkin paste with 5% MSG (*w*/*w*), firstly, and cultured for 1 d. Subsequently, *Lb. plantarum* EJ2014 was added and incubated for 7 d. The co-fermented pumpkin contained 1.47% GABA. *B. subtilis* could synthesize PLP and boost the GAD activity of *Lb. plantarum*. At the same time, *B. subtilis* has strong proteinase activity and offers protein hydrolysate for the growth of *Lb. plantarum.*

The co-cultures of LAB and fungi for GABA production had been reported [114]. Co-cultivation of *Lb. plantarum* K154 and fungus *Ceriporia lacerate* efficiently produced GABA (15.53 mg/mL) and other functional ingredients, such as peptides and polysaccharides. *C. lacerate* produced plenty of exopolysaccharides, protease, cellulose, and α-amylase, which provide rich nutrients for the growth of *Lb. plantarum.* Recently, a two-step fermentation process for GABA production was developed by *Candida rugosa* 8YB and *Lactobacillus futsaii* CS3, the former produces l-glutamic acid, and the latter utilizes l-glutamic acid to produce GABA [115]. Therefore, co-culturing with different strains seems to be a promising way to generate GABA.

## 5. Conclusions

Abundant GABA-producing LAB resources have great potential for developing naturally fermented functional food products. The elucidation of the GABA biosynthetic pathway and its regulation mechanism, and the understanding of biochemical properties of the key enzyme GAD in GABA synthesis, lay the theory to improve GABA production at genetic and metabolic levels. Although some studies have partly clarified molecular mechanisms of GABA biosynthesis, the comprehensive mechanism of GABA biosynthesis, especially the regulatory mechanism, needs further study. A large number of GABA-producing LAB genomes have been published, and complete genome information will deepen the comprehension of GABA metabolic activities of strains at the molecular level. GABA synthesis in LAB has been associated with acid resistance of strains, and it has obvious effects on cell physiology. The knowledge of GABA-producing cell physiology offers references to enhance the GABA production level by means of improving cellular technological properties with physiology-oriented engineering strategies. A number of studies suggested that genetic engineering is an effective approach to promote GABA bioconversion through directed modulation of the GABA metabolic pathway, such as the overexpression of GAD, GABA antiporter GadC and regulator, the inactivation of competitive pathway of GABA synthesis, and the improvement of l-glutamate supply. At the same time, co-culture engineering is also an emerging approach with significant advantages for the production of GABA, especially those that effectively utilize raw materials as the precursors for GABA synthesis and produce a variety of beneficial products. The continued advances in both synthetic biology and genetic engineering will promote GABA production of LAB to meet people’s demand for GABA.

## Figures and Tables

**Table 1:** The enzymatic properties of GAD from different LAB.

Microbe	GAD Length (aa)	Optimal pH	Optimal Temperature (°C)	Predicted Molecular Weight (kDa)	K_m_ (mM)	V_max_	Activators	Reference
*Enterococcus avium* M5 *	467	4.5	55	54.4	3.26 ± 0.21	0.01 mM/min	Mn^2+^, Co^2+^, Ca^2+^, Zn^2+^	[56]
***Enterococcus raffinosus* TCCC11660**	NR	4.6	45	55.0	5.26	3.45 μM/min	Mo^6+^, Mg^2+^	[57]
*Lactobacillus fermentum* * YS2	467	4.5	40	54.1	NR	NR	Ca^2+^, Mg^2+^	[24]
*Lactobacillus zymae* GU240 *	479	4.5	41	53	1.7	0.01 mM/min	NR	[58]
*Lb. brevis* 877G *	468	5.2	45	53	3.6	0.06 mM/min	Ca^2+^	[13]
***Lb. brevis* CGMCC 1306**	479	4.4	37	~62	8.22	6.59 U/mg	NR	[59]
*Lb. brevis* CGMCC 1306 *	479	4.8	48	53.47	10.26	8.86 U/mg	NR	[60]
*Lb. brevis* HYE1 *	469	4.0	55	54.32	4.99	0.224 mM/min	NR	[61]
***Lb. brevis* IFO 12005**	480	4.2	30	54	9.3	NR	NH_4_^+^	[62,63]
***Lb. brevis* NPS-QW-145 GadA**	468	4.8	45	53.49	26.95 ± 2.437	9.16 μM/min	NR	[31]
***Lb. brevis* NPS-QW-145 GadB**	479	4.8	40	53.51	21.39 ± 1.142	32.56 μM/min	NR	[31]
***Lb. hilgardii* MYA-9**	NR	4.6	37	60	NR	NR	Na^+^, NH_4_^+^, Mg^2+^	[64]
***Lb. paracasei* NFRI 7415**	481	5.0	50	54.3	5.0	7.5 μM/min	Ca^2+^, NH_4_^+^	[65]
*Lb. plantarum* ATCC 14917 *	469	4.5	40	53	22.8	24.4 U/mg	NR	[66]
***Lb. plantarum* WCFS1**	469	4.8	60	53.75	20.02	73.33 μM/min	NR	[31]
*Lb. sakei* A156 *	479	5.0	55	54.4	0.045	0.011 mM/min	Ca^2+^, Mn^2+^, Co^2+^, Zn^2+^	[67]
***Lb. sakei* OPK2-59**	479	5.0	30	54.4	NR	NR	Ca^2+^, Fe^3+^, Mg^2+^	[68]
***Lc. lactis* 01-7**	466	4.7	NR	53.93	0.51	NR	NR	[69,70]
***S. salivarius* ssp. *thermophilus* Y2**	459	4.0	40	47	2.3	NR	Ba^2+^	[28]

* recombinant protein in *E. coli*. NR, not reported.

## Supplementary Materials

Supplementary materials can be found at https://www.mdpi.com/1422-0067/21/3/995/s1.

## Abbreviations

CAT	Catalase
GABA	Gamma-aminobutyric acid
GABA-AT	GABA aminotransferase
GAD	Glutamate decarboxylase
GDH	l-glutamate dehydrogenase
GRAS	generally recognized as safe
LAB	lactic acid bacteria
MSG	monosodium glutamate
ODHC	2-oxoglutarate dehydrogenase complex
PLP	pyridoxal-5′-phosphate
SSA	succinic semialdehyde
SSADH	succinate semialdehyde dehydrogenase
TCA	tricarboxylic acid
