# Supplementary material for: Production of Gamma-Aminobutyric Acid from Lactic Acid Bacteria: A Systematic Review
Source: Int J Mol Sci. 2020 Feb 3;21(3):995. doi: 10.3390/ijms21030995 (PMC7037312; doi:10.3390/ijms21030995)
Supplement: Supplementary file 1 [file ijms-21-00995-s001.zip › Supplementary Tables/Supplementary Table 1.docx]

**Table S1.** List of the gamma-aminobutyric acid produce by different microorganisms.

| Microorganism | GABA production (g/L) | References |
| --- | --- | --- |
| *Monascus purpureus* | 7.453 | [1] |
| *Monascus sanguineus* | 15.53 mg/gds | [2] |
| *Pichia anomala* MR-1 | 1.200 | [3, 4] |
| *Rhizopus microsporus* var. *oligosporus* IFO 32002 | 17.4 | [5] |
| *Rhizopus microsporus* var. *oligosporus* IFO 32003 | 15 | [5] |
| *Streptomyces cinereus* Y11 | 4.9 | [6] |

References

1. Wang, J.J; Lee, C.L; Pan, T. M. Improvement of monacolin K, gamma-aminobutyric acid and citrinin production ratio as a function of environmental conditions of *Monascus purpureus* NTU 601. *J. Ind. Microbiol. Biotechnol.* **2003**, *30*, 669-676.
2. Dikshit, R.; Tallapragada, P. Screening and optimization of γ-aminobutyric acid production from *Monascus sanguineus* under solid-state fermentation. *Front. Life Sci.* **2015**, *8(2)*, 172-181.
3. Masuda, K.; Guo, X.F; Uryu, N.; Hagiwara, T.; Watabe, S. Isolation of marine yeasts collected from the Pacific Ocean showing a high production of ɤ-aminobutyric acid. *Biosci. Biotechnol. Biochem.* **2008**, *72 (12)*, 3265-3272.
4. Guo, X.F.; Aoki, H.; Hagiwara, T.; Masuda, K.; Watabe, S. (2009). Identification of high gamma-aminobutyric acid producing marine yeast strains by physiological and biochemical characteristics and gene sequence analyses. *Biosci. Biotechnol. Biochem.* **2009**, *73*, 1527-1534.
5. Aoki, H.; Uda, I.; Tagami, K.; Furuya, Y.; Endo, Y.; Fujimoto, K. The production of a new tempeh like fermented soybean containing a high level of γ-aminobutyric acid by anaerobic incubation with *Rhizopus*. *Biosci. Biotechnol. Biochem.* **2003**, *67(5)*, 1018-1023.
6. Jeng, K.C.; Chen, C.S.; Fang, Y.P. Effect of microbial fermentation on content of statin, GABA, and polyphenols in Pu-erh tea. *J. Agr. Food Chem.* **2007**, *55*, 8787-8792.
