# Supplementary material for: Production of Gamma-Aminobutyric Acid from Lactic Acid Bacteria: A Systematic Review
Source: Int J Mol Sci. 2020 Feb 3;21(3):995. doi: 10.3390/ijms21030995 (PMC7037312; doi:10.3390/ijms21030995)
Supplement: Supplementary file 1 [file ijms-21-00995-s001.zip › Supplementary Tables/Supplementary Table 2.docx]

**Table S2.** List of the gamma-aminobutyric acid produce by LAB isolated from various source.

| Microorganism | Isolation source | GABA production (g/L) | References |
| --- | --- | --- | --- |
| *Enterococcus avium* 9184 | Naturally fermented scallop solution | 3.71 | [1] |
| *Enterococcus avium* G-15 | Carrot Leaves | 115.7 | [2] |
| *Enterococcus avium* M5 | Jeotgals, Korean fermented seafood | 18.47 | [3] |
| *Enterococcus faecium* CFR 3003 | Fermented food | 8.00 | [4] |
| *Enterococcus faecium* GZ2 | Fermented soybean product | 41.87 | [5] |
| *Lactobacillus acidophilus* LMG 9433 | Human | 0.02 | [6] |
| [*Lactobacillus acidophilus*](https://www.sciencedirect.com/topics/pharmacology-toxicology-and-pharmaceutical-science/lactobacillus-acidophilus) BCRC 14079 | Commercial yoghurt | 0.23 | [7] |
| *Lactobacillus acidophilus* YA031 | Fermented food | 4.11 | [8] |
| *Lactobacillus amylovorus* B-4437 | NR | 0.93 | [9] |
| *Lactobacillus brevis* | Paocai | 15.37 | [10] |
| *Lactobacillus brevis* 340G | Kimchi | 7.09 | [11] |
| *Lactobacillus brevis* AN 1–5 | Narezushi (Fermented Fish) | 6.16 | [12] |
| *Lactobacillus brevis* AN 2-2 | Narezushi (Fermented Fish) | 5.58 | [12] |
| *Lactobacillus brevis* AN 3-5 | Narezushi (Fermented Fish) | 4.99 | [12] |
| *Lactobacillus brevis* AN 4-5 | Narezushi (Fermented Fish) | 7.21 | [12] |
| *Lactobacillus brevis* ANP 7-6 | Narezushi (Fermented Fish) | 6.57 | [12] |
| *Lactobacillus brevis* BH2 | Kimchi | 20.00 | [13] |
| *Lactobacillus brevis* BJ20 | Salt-fermented Jot-gal (cod gut) | 2.47 | [14] |
| *Lactobacillus brevis* CECT8181 | Sheep cheese | 0.10 | [15] |
| *Lactobacillus brevis* CECT8182 | Goat cheese | 0.10 | [15] |
| *Lactobacillus brevis* CECT8183 | Goat cheese | 0.10 | [15] |
| *Lactobacillus brevis* CGMCC 1306 | Fresh milk | NR | [16] |
| *Lactobacillus brevis* CRL 1942 | Quinoa sourdough | 25.83 | [17] |
| *Lactobacillus brevis* DPC6108 | Infant feces | 32.32 | [18] |
| *Lactobacillus brevis* E-25 | Sourdough | 1.23 | [19] |
| *Lactobacillus brevis* FPA 3709 | Fish intestine | 2.45 | [20] |
| *Lactobacillus brevis* GABA 057 | NR | 22.99 | [21] |
| *Lactobacillus brevis* GABA100 | Kimchi | 27.6 | [22] |
| *Lactobacillus brevis* HYE1 | Kimchi | 1.93 | [23] |
| *Lactobacillus brevis* IFO-12005 | Kome shochu kasu | 1.05 | [24] |
| *Lactobacillus brevis* JCM 1059^T^ | NR | 2.91 | [25] |
| *Lactobacillus brevis* K203 | Korean kimchi | 44.4 | [26] |
| *Lactobacillus brevis* KA20 | Kimchi | 0.06* | [27] |
| *Lactobacillus brevis* LMG 11437 | Silage | 0.04 | [6] |
| *Lactobacillus brevis* NCL912 | Paocai | 205.8 | [28] |
| *Lactobacillus brevis* NCL912 | Paocai | 35.66 | [29] |
| *Lactobacillus brevis* NPS-QW-145 | Kimchi | 25.83 | [30] |
| *Lactobacillus brevis* NPS-QW-171 | Kimchi | 19.63 | [30] |
| *Lactobacillus brevis* NPS-QW-177 | Kimchi | 24.10 | [30] |
| *Lactobacillus brevis* NPS-QW-193 | Kimchi | 23.33 | [30] |
| *Lactobacillus brevis* NPS-QW-216 | Kimchi | 21.69 | [30] |
| *Lactobacillus brevis* NPS-QW-242 | Kimchi | 22.97 | [30] |
| *Lactobacillus brevis* NPS-QW-255 | Kimchi | 19.07 | [30] |
| *Lactobacillus brevis* NPS-QW-267 | Kimchi | 24.99 | [30] |
| *Lactobacillus brevis* NPS-QW-281 | Kimchi | 23.64 | [30] |
| *Lactobacillus brevis* O52 | Kimchi | 0.05 * | [27] |
| *Lactobacillus brevis* OPK-3 | Korean Kimchi | 84.29^#^ | [31] |
| *Lactobacillus brevis* OPY-1 | Korean Kimchi | 0.42* | [32] |
| *Lactobacillus brevis* PM17 | Cheeses | 0.02 * | [33] |
| *Lactobacillus brevis* RK03 | Fish | 62.52 | [34] |
| *Lactobacillus brevis* SB 109 | Narezushi (Fermented Fish) | 7.33 | [12] |
| *Lactobacillus brevis* TAUL 141 | Cheese | 2.49 | [35] |
| *Lactobacillus brevis* TAUL 174 | Cheese | 1.82 | [35] |
| *Lactobacillus brevis* TAUL 179 | Cheese | 2.31 | [35] |
| *Lactobacillus brevis* TAUL 195 | Cheese | 2.52 | [35] |
| *Lactobacillus brevis* TAUL 69 | Cheese | 1.31 | [35] |
| *Lactobacillus brevis* TAUL 70 | Cheese | 2.44 | [35] |
| *Lactobacillus brevis* TCCC13007 | Pickled vegetables | 61 | [36] |
| *Lactobacillus brevis* TISTR 860 | Traditional Thai vegetables | 4.58 | [37] |
| *Lactobacillus brevis* Y8 | Kimchi | 0.04* | [27] |
| *Lactobacillus buchneri* AN 1-1 | Narezushi (Fermented Fish) | 5.56 | [12] |
| *Lactobacillus buchneri* MS | Kimchi | 0.62* | [38] |
| *Lactobacillus buchneri* SB 21 | Narezushi (Fermented Fish) | 6.65 | [12] |
| *Lactobacillus buchneri* WPZ001 | Chinese Fermented sausages | 129 | [39] |
| *Lactobacillus bulgaricus* CFR 2028 | Dahi | 3.81 | [9] |
| *Lactobacillus bulgaricus* PR1 | Cheese | 0.06 * | [33] |
| *Lactobacillus casei* 2749 | Fermented dairy product | 0.03 * | [40] |
| *Lactobacillus casei* LMG 6904 | Cheese | 0.13 | [6] |
| *Lactobacillus casei* Zhang | Koumiss | 0.68* | [41] |
| *Lactobacillus crispatus* RMK567 | Whole milk | 0.75 | [42] |
| *Lactobacillus curvatus* 2770 | Fermented dairy product | 0.03 * | [40] |
| *Lactobacillus curvatus* 2771 | Fermented dairy product | 0.05 * | [40] |
| *Lactobacillus curvatus* LMG 9198 | Milk | 0.04 | [6] |
| *Lactobacillus curvatus* N-19 | Sourdough | 1.46 | [19] |
| *Lactobacillus farciminis* LMG 9200 | Sausage | 0.03 | [6] |
| *Lactobacillus fermentum* HP3 | Thai fermented foods | 2.11 | [43] |
| *Lactobacillus fermentum* YS2 | Chinese traditional pickled vegetable | 5.15 | [44] |
| *Lactobacillus futsaii* CS3 | Kung-Som | 10.5 * | [45] |
| *Lactobacillus graminis* SC-12 | Sourdough | 0.40 | [19] |
| *Lactobacillus helveticus* B_26_W | Fermented dairy product | 0.02 * | [40] |
| *Lactobacillus helveticus* ND01 | Koumiss | 0.17 | [46] |
| *Lactobacillus helveticus* PR4 | Fermented dairy product | 0.01* | [40] |
| *Lactobacillus mali* LMG 6899 | Apple juice from cider press | 0.06 | [6] |
| *Lactobacillus namurensis* NH2 | Fermented pork | 7.34 | [47] |
| *Lactobacillus otakiensis* L3C1R1 | Azorean cheese | 0.66 | [48] |
| *Lactobacillus paracasei* L3B21K4 | Azorean cheese | 0.58 | [48] |
| *Lactobacillus paracasei* L3B21R2 | Azorean cheese | 0.53 | [48] |
| *Lactobacillus paracasei* L3C21M6 | Azorean cheese | 0.57 | [48] |
| *Lactobacillus paracasei* NFRI 7415 | Fermented fish | 31.14 | [49] |
| *Lactobacillus paracasei* PF6 | Cheese | 0.10 * | [33] |
| *Lactobacillus paracasei* PF6 | Cheese | 0.08* | [40] |
| *Lactobacillus paralimentarius* E-106 | Sourdough | 0.35 | [19] |
| *Lactobacillus paraplantarum* N-15 | Sourdough | 0.67 | [19] |
| *Lactobacillus plantarum* 1 TP | Fermented dairy product | 0.01 * | [40] |
| *Lactobacillus plantarum* BC114 | Sichuan pickle | 3.82 | [50] |
| *Lactobacillus plantarum* BCRC 11697 | N.P. | 0.37 | [7] |
| *Lactobacillus plantarum* C48 | Cheese | 0.07 | [51] |
| *Lactobacillus plantarum* C48 | Cheese | 0.02 * | [33] |
| *Lactobacillus plantarum* CGMCC 1.2437^T^ | Fermented cabbage | 74.37 | [52] |
| *Lactobacillus plantarum* DW12 | Fermented food | 4.00 | [8] |
| *Lactobacillus plantarum* ED-10 | Sourdough | 1.59 | [19] |
| *Lactobacillus plantarum* FC_2_10 | Fermented dairy product | 0.04 * | [40] |
| *Lactobacillus plantarum* IFK-10 | Fermented soy bean | 2.68 | [53] |
| *Lactobacillus plantarum* K154 | Korean kimchi | 0.2 | [54] |
| *Lactobacillus plantarum* L2A21R1 | Azorean cheese | 0.94 | [48] |
| *Lactobacillus plantarum* L2C21E8 | Azorean cheese | 0.46 | [48] |
| *Lactobacillus plantarum* L3C1E8 | Azorean cheese | 0.65 | [48] |
| *Lactobacillus plantarum* LMG 6907 | Pickled cabbage | 0.39 | [6] |
| *Lactobacillus plantarum* MNZ | Fermented food | 0.11 | [55] |
| *Lactobacillus plantarum* NDC75017 | Fermented milk | 3.15* | [56] |
| *Lactobacillus plantarum* NTU 102 | Cabbage pickles | 0.63 | [57] |
| *Lactobacillus plantarum* PU11 | Fermented dairy product | 0.08 * | [40] |
| *Lactobacillus plantarum* SC-9 | Sourdough | 0.51 | [19] |
| *Lactobacillus plantarum* Taj-Apis362 | Honeybee | 0.74 | [58] |
| *Lactobacillus reuteri* LMG 9213 | Adult, intestine | 0.05 | [6] |
| *Lactobacillus rhamnosus* FC_3_6 | Fermented dairy product | 0.02* | [40] |
| *Lactobacillus rhamnosus* GG | Feces | 0.44 | [7] |
| *Lactobacillus rhamnosus* LMG 25859 | Mix of western probiotic products | 0.05 | [6] |
| *Lactobacillus rhamnosus* YS9 | Pickled vegetable | 19.28 | [59] |
| *Lactobacillus rossiae* ED-1 | Sourdough | 1.14 | [19] |
| *Lactobacillus sakei* B2-16 | Kimchi | 68.05 | [60] |
| *Lactobacillus sakei* LMG 18175 | Human, intestine | 0.64 | [6] |
| *Lactobacillus salivarius* CFR 2158 | NR | 1.75 | [9] |
| *Lactobacillus viridescencs* LMG 3507 | Cured meat products | 0.05 | [6] |
| *Lactococcus lactis* 01-7 | Cheese starter | 0.03 | [61] |
| *Lactococcus lactis* CECT 8184 | Goat cheese | 0.10 | [15] |
| *Lactococcus lactis* DIBCA13 | Fermented dairy product | 0.01 * | [40] |
| *Lactococcus lactis* DIBCA2 | Fermented dairy product | 0.06 * | [40] |
| *Lactococcus lactis* NCDO 2118 | Frozen peas | 0.89 | [62] |
| *Lactococcus lactis* PU1 | Cheese | 0.26 * | [63] |
| *Lactococcus lactis* PU1 | Cheese | 0.04 * | [33] |
| *Lactococcus lactis* subsp. *cremoris* LMG 6897 | Cheese starter culture | 0.60 | [6] |
| *Lactococcus lactis* subsp. *lactis* B | Kimchi | 6.41 | [64] |
| *Lactococcus lactis* subsp. *lactis* GE 102 | Cheese | 0.57 | [35] |
| *Lactococcus lactis* subsp. *lactis* GE 103 | Cheese | 0.77 | [35] |
| *Lactococcus lactis* subsp. *lactis* GE 118 | Cheese | 0.52 | [35] |
| *Lactococcus lactis* subsp. *lactis* GE 61 | Cheese | 0.67 | [35] |
| *Lactococcus* sp. 01-4 | Cheese starter | 0.02 | [61] |
| *Lactococcus* sp. 53-1 | Cheese starter | 0.03 | [61] |
| *Lactococcus* sp. 53-7 | Cheese starter | 0.01 | [61] |
| *Leuconostoc mesenteroides* N-6 | Sourdough | 1.57 | [19] |
| *Leuconostoc pseudomesenteroides* N-13 | Sourdough | 1.05 | [19] |
| *Leuconostoc citreum* SC-10 | Sourdough | 0.51 | [19] |
| *Leuconostoc citreum* SC-7 | Sourdough | 0.47 | [19] |
| *Pediococcus damnosus* LMG 11484 | Lager beer yeast | 0.02 | [6] |
| *Pediococcus pentosaceus* HN8 | Fermented beef | 9.06 | [47] |
| *Pediococcus pentosaceus* NH102 | Fermented pork | 8.39 | [47] |
| *Pediococcus pentosaceus* NH116 | Fermented pork | 8.41 | [47] |
| *Pediococcus pentosaceus* IFK-11 | Fermented soy bean | 2.06 | [53] |
| *Pediococcus pentasaceus* LMG 11488 | Dried American beer yeast | 0.06 | [6] |
| *Pediococcus pentosaceus* LMG 10740 | Plants | 0.06 | [6] |
| *Propionibacterium freudenreichii* LMG 16412 | Cheese | 0.07 | [6] |
| [*Streptococcus salivarius*](https://www.sciencedirect.com/topics/pharmacology-toxicology-and-pharmaceutical-science/streptococcus-salivarius) subsp. *thermophilus* BCRC 14085 | Yoghurt | 0.27 | [7] |
| *Streptococcus salivarius* subsp. *thermophilus* Y2 | NR | 7.98 | [65] |
| *Streptococcus thermophilus* APC151 | Fish intestine | 2.2 | [66] |
| *Streptococcus thermophilus* fmb5 | Yogurt | 9.66 | [67] |
| *Streptococcus thermophilus* ST110 | Yogurt starter | 0.66 | [68] |
| *Streptococcus thermophilus* QYW-LYS1 | Fermented yoghurt | 2.91 | [69] |
| *Weissella cibaria* N-9 | Sourdough | 1.27 | [19] |
| *Weissella cibaria* SC-20 | Sourdough | 0.39 | [19] |
| *Weissella hellenica* SB 101 | Fermented foods | 7.18 | [12] |
| *Weissella hellenica* SB 105 | Fermented foods | 7.69 | [12] |
| *Weissella paramesenteroides* N-7 | Sourdough | 1.90 | [19] |

NR, not reported; *, g/Kg; ^#^, mg/L/h.

References

1. Yang, H.; Xing, R.; Hu, L.; Liu, S.; Li, P. Accumulation of γ-aminobutyric acid by *Enterococcus avium* 9184 in scallop solution in a two-stage fermentation strategy. *Microb. Biotechnol.* **2016**, *9(4)*, 478-485.
2. Tamura, T.; Noda, M.; Ozaki, M.; Maruyama, M.; Matoba, Y.; Kumagai, T.; Sugiyama, M. Establishment of an efficient fermentation system of gamma-aminobutyric acid by a lactic acid bacterium.; *Enterococcus avium* G-15.; isolated from carrot leaves. *Biol. Pharm. Bull.* **2010**, *33(10*), 1673-1679.
3. Lee, K.W.; Shim, J.M.; Yao, Z.; Kim, J.A.; Kim, H.J.; Kim, J.H. Characterization of a Glutamate Decarboxylase (GAD) from *Enterococcus avium* M5 Isolated from Jeotgal, a Korean Fermented Seafood. *J. Microbiol. Biotechnol.* **2017**, *27(7)*, 1216-1222.
4. Divyashri, G.; Prapulla, S.G. An insight into kinetics and thermodynamics of gamma-aminobutyric acid production by *Enterococcus faecium* CFR 3003 in batch fermentation. [*Ann. Microbiol*](https://link.springer.com/journal/13213)*.* **2015**, *65(2)*, 1109-1118.
5. Zhu, H.; Sadiq, F.A.; Li, Y.; Yang, S.Y.; Zhou, F. Application of ion-exchange resin as solid acid for buffer-free production of γ-aminobutyric acid using *Enterococcus faecium* cells. *LWT-Food Sci. Technol.* **2018**, *98*, 341-348.
6. Parmentier, N. Screening of gaba producing lactic acid bacteria and increasing the gaba content in soy milk [D]. Ph. D. thesis, University of Gent, **2018**, 1-61.
7. Song, H.Y.; Yu, R.C. Optimization of culture conditions for gamma-aminobutyric acid production in fermented adzuki bean milk. *J. Food Drug Anal.* **2018**, *26*, 74-81.
8. Ratanaburee, A.; Kantachote, D.; Charernjiratrakul, W.; Penjamras, P.; Chaiyasut, C. Enhancement of γ-aminobutyric acid in a fermented red seaweed beverage by starter culture *Lactobacillus plantarum* DW12. *Electron J. Biotechnol.* **2011**, *14(3)*, doi: 10.2225/vol14-issue3-fulltext-2.
9. Gangaraju, D.S.; Murty,V.R.; Prapulla, S.G. Probiotic-mediated biotransformation of monosodium glutamate to γ-aminobutyric acid: differential production in complex and minimal media and kinetic modelling. *Ann. Microbiol.* **2014**, *64(1)*, 229-237.
10. Li, H.; Gao, D.; Cao, Y.S.; Xu, H. A high γ-aminobutyric acid producing *Lactobacillus brevis* isolated from Chinese traditional paocai. *Ann. Microbiol.* **2008**, *58(4)*, 649-653.
11. Seo, M.J.; Lee, Y.J.; Nam, D.Y.; Lee, Y.S.; Park, L.S.; Yi, S.H.; Lee, H.M.; Roh, W.S.; Choi, J.H.; Lim, I.S. Production of γ-aminobutyric acid by *Lactobacillus brevis* 340G isolated from kimchi and its application to skim milk. *Food Eng. Pro*. **2013**, *4*, 418-423.
12. Barla, F.; Koyanagi, T.; Tokuda, N.; Matsui, H.; Katayama, T.; Kumagai, H.; Michihata, T.; Sasaki, T.; Tsuji, A.; Enomoto, T. The γ-aminobutyric acid-producing ability under low pH conditions of lactic acid bacteria isolated from traditional fermented foods of Ishikawa Prefecture.; Japan.; with a strong ability to produce ACE-inhibitory peptides. *Biotechnol. Rep.* **2016**, *10*, 105-110.
13. Kim, S.H.; Shin, B.H.; Kim, Y.H.; Nam, S.W.; Jeon, S.J. Cloning and expression of a full-length glutamate decarboxylase gene from *Lactobacillus brevis* BH2. *Biotechnol. Bioproc. E.* **2007**, 12, 707-712.
14. Lee, B.J.; Kim, J.S.; Kang, Y.M.; Lim, J.H.; Kim, Y.M.; Lee, M.S.; Jeong, M.H.; Ahn, C.B.; Je, J.Y. Antioxidant activity and γ-aminobutyric acid (GABA) content in sea tangle fermented by *Lactobacillus brevis* BJ20 isolated from traditional fermented foods. *Food Chem.* **2010**, *122 (1)*, 271-276.
15. Diana, M.; Tres, A.; Quilez, J.; Llombart, M.; Rafecas, M. Spanish cheese screening and selection of lactic acid bacteria with high gamma-aminobutyric acid production. *LWT-Food Sci Technol.* **2014**, *56*, 351-355.
16. Huang, J.; Mei, L.H.; Sheng, Q.; Yao, S.J.; Lin, D.Q. Purification and characterization of glutamate decarboxylase of *Lactobacillus brevis* CGMCC 1306 isolated from fresh milk. *Chinese J. Chem. Eng.* **2007**, *15*, 157-161.
17. Villegas, J.M.; Brown, L.; Hebert, E.M.; et al. Optimization of batch culture conditions for GABA production by *Lactobacillus brevis* CRL 1942, isolated from quinoa sourdough. *Food Sci. Technol.* **2016**, *67*, 22-26.
18. Barrett, E.; Ross, R.P.; O'Toole, P.W.; Fitzgerald, G.F.; Stanton, C. Gamma-aminobutyric acid production by culturable bacteria from the human intestine. *J. Appl. Microbiol.* **2014**, *113(2)*, 411-417.
19. Demirbaş, F.; İspirli, H.; Kurnaz, A.A.; Yılmaz, M.T.; Dertli, E. Antimicrobial and functional properties of lactic acid bacteria isolated from sourdoughs. *LWT-Food Sci Technol.* **2017**, *79*, 361-366.
20. Ko, C.Y.; Lin H.T.V.; Tsai, G J. Gamma-aminobutyric acid production in black soybean milk by *Lactobacillus brevis* FPA 3709 and the antidepressant effect of the fermented product on a forced swimming rat model. *Process Biochem.* **2013**, *48*, 559-568.
21. Choi, S.I.; Lee, J.W.; Park, S.M.; Lee, M.Y.; Ji, G.E.; Park, M.S.; Heo TR. Improvement of γ-aminobutyric acid (GABA) production using cell entrapment of *Lactobacillus brevis* GABA 057. *J. Microbiol. Biotechnol.* **2006**, *16(4)*, 562-568.
22. Kim, J.Y.; Lee, M.Y.; Ji, G.E.; Lee, Y.S.; Hwang, K.T. Production of gamma-aminobutyric acid in black raspberry juice during fermentation by *Lactobacillus brevis* GABA100. [*Int. J. Food Microbiol.*](https://www.ncbi.nlm.nih.gov/pubmed/19167126) **2009**, *130(1)*, 12-16.
23. Lim, H.S.; Cha, I.T.; Roh, S.W.; Shin, H.H.; Seo, M.J. Enhanced production of gamma-Aminobutyric acid by optimizing culture conditions of *Lactobacillus brevis* HYE1 isolated from Kimchi, a Korean fermented food. *J. Microbiol. Biotechnol.* **2017**, *27(3)*, 450-459.
24. Yokoyama, S.; Hiramatsu, J.; Hayakawa, K. Production of gamma-aminobutyric acid from alcohol distillery lees by *Lactobacilus brevis* IFO- 12005. *J. Biosci. Bioeng.* **2002**, *93(1)*, 95-97.
25. Hasegawa, M.; Yamane, D.; Funato, K.; Yoshida, A.; Sambongi, Y. Gamma-aminobutyric acid fermentation with date residue by a lactic acid bacterium.; *Lactobacillus brevis*. *J. Biosci. Bioeng.* **2018**, *125(3)*, 316-319.
26. Binh, T.T.; Ju, W.T.; Jung, W.J.; Park, R.D. Optimization of gamma-amino butyric acid production in a newly isolated *Lactobacillus brevis*. *Biotechnol.. Lett.* **2014**, *36*, 93-98.
27. Yu, H.H.; Choi, J.H.; Kang, K.M.; Hwang, H.J. Potential of a lactic acid bacterial starter culture with gamma-aminobutyric acid (GABA) activity for production of fermented sausage. *Food Sci. Biotechnol.* **2017**, *26(5)*, 1333-1341.
28. Wang, Q.; Liu, X.; Fu, J.; Wang, S.; Chen, Y.; Chang, K.; Li, H. Substrate sustained release-based high efficacy biosynthesis of GABA by *Lactobacillus brevis* NCL912. *Microb. Cell Fact.* **2018**, *17(1)*, 80.
29. Li, H.; Qiu, T.; Gao, D.; Cao, Y. Medium optimization for production of gamma-aminobutyric acid by *Lactobacillus brevis* NCL912. *Amino Acids* **2010**, *38*, 1439-1445.
30. Wu, Q.; Shah, N.P. Gas release-based prescreening combined with reversed-phase HPLC quantitation for efficient selection of highgamma-aminobutyric acid (GABA)-producing lactic acid bacteria. *J. Dairy Sci.* **2015**, 98, 790-797.
31. Park, K.B.; Oh, S.H. Cloning.; sequencing and expression of a novel glutamate decarboxylase gene from a newly isolated lactic acid bacterium.; *Lactobacillus brevis* OPK-3. *Bioresource Technol.* **2007**, *98*, 312-319.
32. Park, K.B.; Oh, S.H. Production of yogurt with enhanced levels of gamma-aminobutyric acid and valuable nutrients using lactic acid bacteria and germinated soybean extract. *Bioresource Technol.* **2007**, *98*, 1675-1679.
33. Siragusa, S.; de Angelis, M.; di Cagno, R.; Rizzello, C.G.; Coda, R.; Gobbetti, M. Synthesis of γ-aminobutyric acid by lactic acid bacteria isolated from a variety of Italian cheeses. *Appl. Environ. Microbiol.* **2007**, *73*, 7283-7290.
34. Wu, C.H.; Hsueh, Y.H.; Kuo, J.M.; Liu, S.J. Characterization of a potential probiotic *Lactobacillus brevis* RK03 and efficient production of γ-Aminobutyric acid in batch fermentation. *Int. J. Mol. Sci.* **2018**, *19(1)*, 143.
35. Renes, E.; Linares, D.M.; González, L.; Fresno, J.M.; Tornadijo, M.E.; Stanton, C. Production of conjugated linoleic acid and gamma-aminobutyric acid by autochthonous lactic acid bacteria and detection of the genes involved. *J. Funct. Foods* **2017**, *34*, 340-346.
36. Zhang, Y.; Song, L.; Gao, Q.; Yu, S.M.; Li, L.; Gao, N.F. The two-step biotransformation of monosodium glutamate to GABA by *Lactobacillus brevis* growing and resting cells. *Appl. Microbiol. Biot.* **2012**, *94*, 1619-1627.
37. Saraphanchotiwitthaya, A.; Sripalakit, P. Production of γ-aminobutyric acid from red kidney bean and barley grain fermentation by *Lactobacillus brevis* TISTR 860. *Biocatal. Agric. Biotechnol.* **2018**, *16*, 49-53.
38. Cho SY.; Park MJ.; Kim KM.; Ryu JH.; Park HJ. Production of high γ-aminobutyric acid (GABA) sour kimchi using lactic acid bacteria isolated from mukeunjee kimchi. *Food Sci. Biotechnol.* **2011**, *20 (2)*, 403-408.
39. Zhao, A.; Hu, X.; Pan, L.; Wang, X. Isolation and characterization of a gamma-aminobutyric acid producing strain *Lactobacillus buchneri* WPZ001 that could efficiently utilize xylose and corncob hydrolysate. *Appl. Microbiol. Biot.* **2015**, *99*, 3191-3200.
40. Nejati, F.; Rizzello, C.D.; Cagno, R.; Sheikh-Zeinoddin, M.; Diviccaro, A.; Minervini, F.; Gobetti, M. Manufacture of a functional fermented milk enriched of angiotensin-I converting enzyme (ACE)-inhibitory peptides and gamma amino butyric acid (gaBa). *LWT-Food Sci. Technol.* **2013**, *51 (1)*, 183-189.
41. Wang, H.K.; Dong, C.; Chen, Y.F.; Cui, L.M.; Zhang, H.P. A new probiotic Cheddar cheese with high ACE-inhibitory activity and gamma-aminobutyric acid content produced with Koumiss-derived *Lactobacillus casei* Zhang. *Food Technol. Biotech.* **2010**, *48*, 62-67.
42. Oh, N.R. Physiological characteristics of *Lactobacillus cripatus* RMK 567 isolated from milk and optimization of GABA producing condition. Master thesis.; Sungkyunkwan Univ.; Suwon.; Korea.; 2006.
43. Woraharn, S.; Lailerd, N.; Sivamaruthi, B.S.; Wangcharoen, W.; Sirisattha, S.; Peerajan, S.; Chaiyasut, C. Evaluation of factors that influence the L-glutamic and γ-aminobutyric acid production during *Hericium erinaceus* fermentation by lactic acid bacteria. *CyTA-J. Food* **2016**, *14(1)*, 47-54.
44. Lin, Q.; Li, D.N.; Qin, H.Z. Molecular cloning, expression, and immobilization of glutamate decarboxylase from *Lactobacillus fermentum* YS2. *Electron J. Biotechn.* **2017**, *27*, 8-13.
45. Sanchart, C.; Rattanaporn, O.; Haltrich, D.; Phukpattaranont, P.; Maneerat, S. Enhancement of gamma-aminobutyric acid (GABA) levels using an autochthonous *Lactobacillus futsaii* CS3 as starter culture in Thai fermented shrimp (Kung-Som) . W*orld J. Microbiol. Biotechnol.* **2017**, *33*, 152.
46. Sun, T.S.; Zhao, S.P.; Wang, HK.; Cai, C.K.; Chen, Y.F.; Zhang, H.P. ACE-inhibitory activity and gamma-aminobutyric acid content of fermented skim milk by *Lactobacillus helveticus* isolated from Xinjiang koumiss in China. *Eur. Food Res. Technol.* **2009**, *228*, 607-612.
47. Ratanaburee, A.; Kantachote, D.; Charernjiratrakul, W.; Sukhoom, A. Selection of gamma-aminobutyric acid-producing lactic acid bacteria and their potential as probiotics for use as starter cultures in Thai fermented sausages (Nham). *Int. J. Food Sci. Tech.* **2013**, *48*, 1371-1382.
48. Ribeiro, S.C.; Domingos‐Lopes, M.F.P.; Stanton, C.; Ross, R.P.; Silva C.CG. Production of γ‐aminobutyric acid (GABA) by *Lactobacillus otakiensis* and other *Lactobacillus* sp. isolated from traditional pico cheese. *Int. J. Dairy Technolo.* **2018**, *71(4)*, 1012-1017.
49. Komatsuzaki, N.; Shima, J.; Kawamoto, S.; Momose, H.; Kimura, T. Production of gamma-aminobutyric acid (GABA) by *Lactobacillus paracasei* isolated from traditional fermented foods. *Food Microbiol.* **2005**, *22*, 497-504.
50. Zeng, L.; Tan, X.; Zhang, Q.; Yang, Y.; Tang, J. Optimization of γ-aminobutyric acid production by *Lactobacillus plantarum* BC114 from Sichuan pickle. *Food Fermentation Industries*.2**017**, *43(3)*, 116-22. (In Chinese)
51. Servili, M.; Rizzello, G.C.; Taticchi, A.; Esposto, S.; Urbani, S.; Mazzacane, F.; Maio, D.; Selvaggini, I.; Gobbettib, M.; Cagno, D.R. Functional milk beverage fortified with phenolic compounds extracted from olive vegetation water.; and fermented with functional lactic acid bacteria. *Int. J .Food Microbiol.* **2011**, *147*, 45-52.
52. Zhuang, K.; Jiang, Y.; Feng, X.; Li, L.; Dang, F.; Zhang, W.; Man, C. Transcriptomic response to GABA-producing *Lactobacillus plantarum* CGMCC 1.2437^T^ induced by L-MSG. *PLoS ONE* **2018**, *13(6)*, e0199021.
53. Agung, Y.I.B.; Kusumawati, I.G.A.W.; Sumadewi, N.L.U.; Rahayu, E.S.; Indrati R. Isolation and identification of lactic acid bacteria from Indonesian fermented foods as γ-aminobutyric acid-producing bacteria. *Int. Food Res. J.* **2018**, *25 (4)*, 1753-1757.
54. Park, S.Y.; Lee, J.W.; Lim, S.D. The probiotic characteristics and GABA production of *Lactobacillus plantarum* K154 isolated from kimchi. *Food Sci. Biotechnol.* **2014**, *23*, 1951-1957.
55. Zareian, M.; Ebrahimpour, A.; Bakar F.A.; Mohamed, A.K.; Forghani, B.; Ab-Kadir. M.S; Saari, N. A glutamic acid-producing lactic acid bacteria isolated from Malaysian fermented foods. *Int. J. Mol .Sci.* **2012**, *13*, 5482-5497.
56. Shan, Y.; Man, C.X.; Han, X.; Li, L.; Guo, Y.; Deng, Y.; Li, T.; Zhang, L.W.; Jiang, Y.J. Evaluation of improved gamma-aminobutyric acid production in yogurt using *Lactobacillus plantarum* NDC75017. *J. Dairy Sci.* **2015**, *98(4)*, 2138-2149.
57. Tung, Y.; Lee, B.H.; Liu, C.F.; Pan, T.M. Optimization of culture condition for ACEI and GABA production by lactic acid bacteria. *J. Food Sci.* **2011**, *76*, 585-591.
58. Tajabadi, N.; Ebrahimpour, A.; Baradaran, A.; Rahim, R.A.; Mahyudin, N.A.; Manap, M.Y.; Bakar, F.A.; Saari, N. Optimization of γ-aminobutyric acid production by *Lactobacillus plantarum* Taj-Apis362 from honeybees. *Molecules*, **2015**, *20*, 6654-6669.
59. Lin, Q. Submerged fermentation of *Lactobacillus rhamnosus* YS9 for gamma-aminobutyric acid (GABA) production. *Braz, J. Microbiol.* **2013**, *44*, 183-187.
60. Seo, Y.C.; Choi, W.Y.; Kim, K.S.; Lee, C.G.;Ahn, J.H .; Cho, H.Y.; Lee, S.H.; Cho, J.S.; Joo, S.J.; Lee, H.Y. Enhancement of the cognitive effects of γ-Aminobutyric acid from monosodium glutamate fermentation by *Lactobacillus sakei* B2-16. *Food Biotechnol.* **2012**, *26*, 29-44.
61. Nomura, M.; Kimoto, H.; Someya, Y.; Furukawa, S.; Suzuki, I. Production of gamma-aminobutyric acid by cheese starters during cheese ripening. *J. Dairy Sci.* **1998**, *81(6)*, 1486-1491.
62. Laroute, V.; Yasaro, C.; Narin, W.; Mazzoli, R.; Pessione, E.; Cocaign-Bousquet, M.; Loubière, P. GABA production in *Lactococcus lactis* is enhanced by arginine and co-addition of malate. *Front. Microbiol.* **2016**, *7*, 1050.
63. Rizzello, C.G.; Cassone, A.; Cagno, R.D.I.; Gobbetti, M. Synthesis of Angiotensin I-Converting Enzyme (ACE)-Inhibitory peptides and γ-aminobutyric acid (GABA) during sour dough fermentation by selected lactic acid bacteria. *J. Agr. Food Chem.* **2008**, *56*, 6936-6943.
64. Lu, X.; Chen, Z.; Gu, Z.; Han, Y. Isolation of γ-aminobutyric acid producing bacteria and optimization of fermentative medium. *Biochem. Eng. J.* **2008**, *41*, 48-52.
65. Yang, S.Y.; Lu, F.X.; Lu, Z.X.; Bie, X.M.; Jiao, Y.; Sun, L.J.; Yu, B. Production of γ-aminobutyric acid by *Streptococcus salivarius* subsp. *thermophilus* Y2 under submerged fermentation. *Amino acids***2008**, *34(3)*, 473-478.
66. Linares, D.M.; O'Callaghan, T.F.; O'Connor, P.M.; Ross, R.P.; Stantonm C. *Streptococcus thermophilus* APC151 strain is suitable for the manufacture of naturally GABA-enriched bioactive yogurt food. *Front. Microbiol.* **2016**, *7*, 1876.
67. Chen, L.; Alcazar, J.; Yang, T.; Lu, Z.; Lu, Y. Optimized cultural condition of function yogurt for $\gamma$-aminobutyric acid augmentation using response surface methodology. *J. Dairy Sci.* **2018**, *101*, 1-9.
68. Somkuti, G.A.; Renye, J.A.; Steinberg, D.H. Molecular analysis of the glutamate decarboxylase locus in *Streptococcus thermophilus* ST110. *J. Ind. Microbiol. Biot.* **2012**, *39*, 957-963.
69. Liu, H.; Zhang, J.; Yang, W.Q.; Liu, Z.Y.; Chen, X.; Guo, H.; Liu, Y.B.; Zhang, C.G.; Liu, Y.C.; Liu, J.C.; Guan, H. Bio-synthesis of GABA by *Streptococcus thermophilus* QYW-LYS1 isolated from traditional fermented yoghurt. Adv. Mater. Res. **2014**, *884-885*, 401-404.
